# Comparison of the osteogenic capability of rat bone mesenchymal stem cells on collagen, collagen/hydroxyapatite, hydroxyapatite and biphasic calcium phosphate

**DOI:** 10.1093/rb/rbx018

**Published:** 2017-06-30

**Authors:** Xiaoyu Sun, Wen Su, Xiaomin Ma, Huaiying Zhang, Zhe Sun, Xudong Li

**Affiliations:** National Engineering Research Center for Biomaterials, Sichuan University, Chengdu 610064, P.R. China

**Keywords:** osteogenic differentiation, spreading morphology, mineralization of collagen, bioceramics

## Abstract

Collagen (COL), collagen/hydroxyapatite (COL/HA), HA and biphasic calcium phosphate were prepared as representative bone grafting materials with composition analogous to bone, and their structural characteristics were analyzed. The rat bone mesenchymal stem cells (BMSCs) were further seeded onto four groups of materials, and BMSCs grown in basic medium and standard osteogenic medium were set as controls of a reference model to show the basic and osteogenic behavior of cells without the intervention of materials. Cellular behaviors were characterized, including proliferation, spreading morphology and expression of osteogenesis factors. The rat BMSCs proliferated properly with time on four groups of materials as well on two groups of controls, and typical cuboidal, polygonal and extremely-elongated morphologies of cells were observed. According to the real-time polymerase chain reaction data, a higher osteogenic gene expression level was dependent upon the growing morphology but not the proliferation rate of cells, and the osteogenic differentiation capacity of cells onto four groups of materials varied in specific genes. In general, BMSCs exhibited the highest osteogenic capacity onto COL/HA, but the poorest onto HA. The growing behaviors of cells on materials were further discussed in comparison with the cases of OC and BC of the reference model. The present attempt to comparatively analyze cell experimental data with a reference model is expected to be useful for revealing the difference in the osteogenic capability of MSCs onto materials or even the bioactivity of materials.

## Introduction

Development of biomaterials with improved properties for bone tissue substitution has being a key theme in the field of biomedical engineering and regenerative medicine [1, 2]. Owing to various congenital, degenerative and ever-increasing traumatic conditions, the cases of bone defects required for surgical intervention increase in number each year. As bone tissue has only limited ability to regenerate, the use of bone graft substitutes is thus indispensable. Autologous graft is known as the gold standard in the bone replacement, but is concomitant with shortcomings such as limited graft quantity and donor site morbidity [3]. Accordingly, various synthetic bone graft substitutes including metallic implants [4], bioactive glasses [5] and ceramics [6, 7], and bone-like composites [8, 9], have been studied in order to meet the clinical need for reconstruction of bone deficiencies. Systematic *in vitro* and *in vivo* assessments are generally involved in these studies. In the *in vitro* aspects, a variety of advanced analytical and testing techniques have been applied to characterize physicochemical and biological properties of bone graft materials. These *in vitro* measurements provide important supports for understanding the *in vivo* performances of a specific material post-implantation, and also a wealth of such *in vitro* data facilitate to comparatively analyze the difference in the bioactivity of materials. Nowadays, with the development of molecular biology and cell culture techniques, the growth behaviors of cells seeded on materials have received unprecedented attentions. Among different cell lineages for cell culture, mesenchymal stem cells (MSCs) are popular for investigating the cell viability and osteogenic differentiation as well as directly for developing MSC-cultured constructs for bone tissue engineering purposes [10, 11].

MSCs have the potential to differentiate along different lineages including those of bone (osteoblasts), cartilage (chondrocytes), fat (adipocytes) and muscle (myocytes) [12]. This capability for specific lineage differentiation mentioned earlier is governed at least by geometric cues [13, 14], material-dependent factors [15–20] and chemical agents [21–23]. The osteogenic differentiation of MSCs is certainly associated with studies of characterizing various bone graft substitutes. In general, cells were cultured onto materials in the absence or presence of chemical agents, and several markers of osteogenesis such as runt-related transcription factor 2 (RUNX-2), alkaline phosphatase (ALP), osterix (OSX), collagen type I (COL-I), osteocalcin (OCN), bone sialoprotein and osteopontin were analyzed at different culturing intervals [6]. In most cases, such studies were only paid to investigating the influence of materials, either one or a combination of different materials, including COL glycosaminoglycan scaffold [24], biphasic calcium phosphate (BCP) ceramics [25, 26], hydroxyapatite (HA) and β-tricalcium phosphate (β-TCP) [6, 27, 28], HA and mineralized COL [29], HUVEC-derived ECM and β-TCP [30], and COL, COL/HA and HA [31, 32]. These studies not only reported the actual results about the proliferation and osteogenic differentiation of cells on a specific material, but also stated the relevant differences among materials.

Studies show that chemical agents including dexamethasone (Dex), ascorbic acid and β-glycerophosphate were beneficial to the osteogenic differentiation of MSCs [33–35]. The extensive *in vitro* studies above have had a major impact upon our knowledge of the osteogenic behaviors of stem cells on materials in the absence or presence of chemical agents. However, it is worthy to note that a systematic comparative analysis of cell culturing data has received much less attention until now. The reported results in most cases of previous cell culturing studies were merely descriptive, due to the absence of a reference model as a foil for comparing the osteogenic capacity of MSCs. It is believed that both basic/normal and osteogenic behaviors of cells without the intervention of any materials should be taken into account for constituting such a reference model. In fact, this could be realized by culturing of MSCs on tissue culture (TC) plate both in basic medium (BM) and in osteogenic medium which gives rise to two groups of controls, i.e. basic control (BC) and osteogenic control (OC). The osteogenic medium often contains 100 nM Dex, 10 mM β-glycerophosphate and 50 µM L-ascorbic acid in BM. The selection of this osteogenic medium was based on the widely accepted promoting effects of a standard combination of Dex, β-glycerophosphate and L-ascorbic acid [12, 24, 33, 35].

Hard tissues such as bone and tooth are known as naturally occurring organic/mineral nanocomposites. The organic component is mainly fibrillar COL-I, and the mineral phase is nano-sized calcium phosphates [36]. Therefore, monomeric COL or fibrillar COL have been studied and applied in clinical treatment as bone graft substitutes [37–40]. In addition, representative (CaP)-based ceramics, HA, TCP and BCP, have been extensively investigated for their ‘intrinsic’ osteoinduction property [25, 41, 42], and the BCP revealed better osteogenesis results among these ceramics [6]. Recently, the composite of COL and HA have been reported superb osteogenesis effect *in vitro* [29, 43–45]. In this study, four groups of materials were prepared as representative constituents of bone, and they were COL, COL/HA, HA and BCP. The COL-based materials were obtained by using self-assembly of COL in the absence and presence of simultaneous HA synthesis, and were thus fibrillar COL and mineralized COL. Both HA and BCP were sintered bioceramic samples. The rat bone MSCs (BMSCs) were seeded on four groups of bone substitutes, and cells grown in BM and osteogenic medium were used as the BC and OC of a reference model without the intervention of any materials. The proliferation, spreading morphology and osteogenic differentiation of rat BMSCs cultured on four groups of materials together with two groups of controls for up to 28 days were characterized. This attempt to compare rate of the osteogenic capability of BMSCs among materials with an aid of a reference model is expected to be useful for understanding the differences in biological performances of biomaterials.

## Materials and methods

### Materials and reagents

Type I COL was extracted from calf skin by pepsin digestion, and dissolved in 0.3 M acetic acid solution at 4°C to obtain the COL acidic solution (8 mg/ml) for this study [38]. NaOH and acetic acid were purchased from Kelong Chemical Co. (Chengdu, China). CaCl_2_, Ca(NO_3_)_2_, (NH_4_)_2_HPO_4_ and NaH_2_PO_4_·2H_2_O were purchased from Sigma-Aldrich Co. LLC. (MO, USA). Phosphate buffer saline (PBS) was provided by Solarbio Life Sciences (Beijing, China). α-Minimum Essential Medium (α-MEM, Hyclone), fetal bovine serum (Gibco), trypsin (Hyclone), penlcillin-streptomycin solution (Hyclone), Dex (Sigma), β-glycerophosphate (Sigma) and L-ascorbic acid (Sigma) were all used in cell culture experiments. Fluorescein diacetate (FDA), tetramethylrhodamine (TRITC)-phalloidin and Hoechst 33342 were purchased from Sigma-Aldrich (St Louis, MO). The DNA primers of β-actin, ALP, COL-I, OCN, RUNX-2 and OSX were synthesized by Tiantai Life Science (Chengdu, China).

### Preparation of bone graft substitutes

Four kinds of bone graft substitutes, COL, COL/HA, HA and BCP, were prepared on the basis of the constituents of natural bone. *In vitro* COL reconstitution was employed to prepare COL-based substitutes whereas HA and BCP were both the sintered bioceramic products.

COL samples: At 4 °C, the pH of COL solutions was adjusted by using NaOH and acetic acid to 7.20, and then adding 0.3 M NaH_2_PO_4_ making the final phosphate concentration up to 20 mM. Subsequently, 100 µl of the neutral COL solutions was paved onto each well of 24-wells plate or a TC-treated glass coverslip. The reconstitution of COL into COL fibrils was triggered by elevating the temperature from 4 to 37 °C. Finally, COL coated wells or coverslips were carefully washed with deionized water for the cell experiments.

#### COL/HA samples

In the acidic COL solutions, Ca^2+^-containing and phosphate aqueous solutions with the Ca/*P* = 1.67 were dropwise added with vigorously stirring, and the ratio of COL/HA was 40/60 (by weight). Then, the pH value of the resultant slurry was adjusted with NaOH to 7.2, and the slurry was kept stirring for 2 h. Subsequently, 100 µl of the reactive slurry was paved onto each well of 24-wells plate or a TC treated glass coverslip. The above procedures were conducted at 4 °C. To achieve COL fibrillogenesis, the 24-wells plate and glass coverslips were moved to a water incubator of 37 °C for 24 h. Finally, COL/HA-coated wells of culture plates and coverslips were carefully washed with deionized water, the ion concentrations in supernatant were monitored by conductometric measurements.

#### HA samples

The wet chemistry of Ca(NO_3_)_2_ and (NH_4_)_2_HPO_4_ solutions with ammonia was applied to synthesize calcium phosphate powder. For HA, the Ca/P ratio of the Ca^2+^-containing solution and phosphate solution was 1.67. The HA powder disks was uniaxially pressed and sintered at 1100°C for 3 h. HA ceramic disks of 12 mm in diameter and 2 mm in thickness were used for this study.

#### BCP samples

The synthesis and sintering procedures for BCP ceramics were identical to the preparation of HA samples. In this study, the ratio of HA to β-TCP was 60/40 in BCP ceramic samples.

### Characterization of synthetic bone substitutes

The chemical species of the prepared samples were analyzed by using Fourier transform infrared spectroscopy (FT-IR, PerkinElmer Spectrum One B System). The powdered samples were used to obtain the KBr pellet. The spectrum in the range of 400–4000 cm^−1^ was collected with 20 scans at a scanning resolution of 4 cm^−1^. The phase and composition of synthetic bone substitutes were measured on a DX-1000 X-ray diffractometer (XRD) with Cu Kα radiation (λ = 1.5406 Å) at the working voltage and current of 40 kV and 25 mA. The XRD data in the 2θ range of 10°−70° were collected at a scanning rate of 0.06° s^−^^1^. The morphology and microstructure of synthetic materials were examined with a field emission scanning electron microscope (FE-SEM, Hitachi S-4800). For FE-SEM observations, the COL-based samples were sputter-coated with gold for 30 s. Before sputter coating, fixation with glutaraldehyde, dehydration using gradient alcohol and subsequent critical point drying were applied to COL and COL/HA samples.

### Isolation and culture of rat BMSCs

The MSCs were isolated from femurs and tibias of rats, ∼1-week old, provided from the experiental animals service of Dossy (Chengdu, China). The procedure was conducted in accordance with the Guidelines for Animal Experimentation (National Engineering Research Center for Biomaterial, Sichuan University). Briefly, the rats were sacrificed by over anesthesia and disinfected in 0.1% bromogeramine and then in 75% alcohol. Skins and soft tissues were removed to expose the long bones. Subsuently, the bone marrow was flushed out by 1 ml injection syringes and dispersed to a culture dish containing BM, consisting of α-MEM, 10% fetal bovine serum, 100 U mL^−1^ penlcillin and 100 U mL^−1^ streptomycin. The BM was changed at 24 h in order to get rid of the non-adherent cells. The cells were passaged using 0.05% trypsin/EDTA when 80% confluence was reached. The phenotype of the isolated cells was identified by analyzing the specifice surface markers with flow cytometry [25]. The third passage (P3) of rat BMSCs were used in this study.

Four kinds of bone graft substitutes, COL/HA, COL, HA and BCP, were placed in polystyrene culture plates and prewetted in α-MEM without serum overnight in the 37 °C incubator. According to the specific purpose of this study, cells at a specific density were seeded onto four groups of material samples, and cultured for up to 28 days. In addition to the BM for cell culturing above, osteogenic medium was also prepared by adding 100 nM Dex, 10 mM β-glycerophosphate and 50 µM L-ascorbic acid in BM. Accordingly, culturing of rat BMSCs in the culture medium alone gave rise to two groups of controls as a reference model, BC and OC, which separately represent the growth of rat BMSCs in BM and in osteogenic medium. The proliferation, spreading morphology and osteogenic differentiation of cells in BC and OC were supportive for comparing cellular behaviors onto four groups of prepared materials. The respective culture medium was changed every three days. All the cell culturing experiments were performed in a 5% CO_2_ humid atmosphere at 37 °C.

### Cell proliferation

The proliferation of rat BMSCs onto COL/HA, COL, HA, BCP and both BC and OC were determined by using MTT (3-(4, 5-dimethylthiazol-2-yl)-2,5-diphenyltetrazolium bromide) reagent dissolved in PBS at 5 mg/ml. Specifically, P3 rat BMSCs were seeded in 24-well plates at 5 × 10^3^ cells per well and were cultured for 1, 4, 7, and 14 days. Samples were incubated with 200 µl MTT reagent at 37 °C in dark for 4 hours, then the mixed medium was replaced by 1 ml of dimethylsulfoxide and the plates were shaken on an oscillator in low frequence for 10 min. Finally, the solutions containing the formazan produced by metabolically active cells reacting with the tetrazolium salt in the MTT reagent were transferred to 96-well plates. The proliferation of the cells was reflected by the optical density measured on a Varioskan Flash Microplate Reader (Thermo Scientific) at an absorbance of 490 nm.

### Cell spreading morphology

The spreading morphology of rat BMSCs cultured in different groups was also revealed by FE-SEM. 5 × 10^3^ cells per sample were initially seeded in 24-well plates and cultured for 4 days before test. After being washed twice by PBS, the samples were fixed by 2.5% glutaraldehyde solution for 12 h at 4 °C and the subsequent procedures were the same as described in ‘Characterization of synthetic bone substitutes’ Section.

After culturing of 4 and 14 days, the state of living rat BMSCs in each group were revealed by FDA staining. Before staining, the samples were washed by PBS slightly and immersed in working solution (10 µg/ml) for 5 min, and then they were rinsed in PBS to remove the residual reagent. Finally, they were observed by confocal laser scanning microscope (CLSM, TCS-SP5, Leica).

In order to visualize actin cytoskeleton and nuclei of the rat BMSCs, samples were subject to the fluorescent staining of TRITC-phalloidin and Hoechst 33342 after culturing of 4 days with initial cell density of 5 × 10^3^ per well in 24-well plates, respectively. In detail, the samples were washed by PBS. Then, they were fixed by 4% paraformaldehyde for 10 mins at 4°C and washed again by PBS. Cells were permeabilized with 0.1% TritonX-100 (Sigma) and blocked by 0.1% BSA/PBS. 200 µl of TRITC-phalloidin (10 µg/ml) was added to the each sample and incubated at 37 °C for 1 h after which they were washed and stained by Hoechst 33342 (10 µg/ml) for 5 min. After washing with 0.1% BSA/PBS, cells were imaged using CLSM.

### Expression of osteogenic genes

#### Isolation of RNA and reverse transcription

To analyze gene expression profiles of specific osteogenic genes, cells were cultured in different groups for 1, 4, 7, 14, 21 and 28 days with primary seeding density of 10^4^ cells per well in 24-well plates. The total RNA was isolated using the Total RNA Miniprep Kit (Axygen) in accordance with the manufacturer’s protocol. After isolation, the concentration of total RNA was quantified by a spectrophotometer (Nanodrop Technologies). In the reverse transcription progress, PrimeScriptTM RT reagent Kit (TaKaRa) was employed to complete the cDNA synthesis.

#### Real-time polymerase chain reaction

The manufacturer’s instruction of SYBR^®^ Premix Ex TaqTM II (TaKaRa) was strictly performed. First, 25 µl reaction system was mixed by 12.5 µl Premix Ex TaqTM II agent, 1 µl forward primer, 1 µl reverse primer, 2 µl cDNA and 8.5 µl sterile deionized water. Second, the polymerase chain reaction initiated with a denaturation step at 95°C for 30 s, followed by 40 cycles of 95°C for 5 s and 60°C for 30 s. This process was carried out in a CFX96TM Real-Time cycler (Bio-Rad). The obtained data were normalized using the Bio-Rad CFX Manager software to calculate relative expressions. The genes detected in this study were RUNX2, OSX, ALP, COL-I and OCN, β-actin was introduced as housekeeping gene. The sequences of applied primers are given in Table 1.

**Table 1:** Sequence of primers used for RT-PCR analysis

Target	Forward primer	Reverse primer
Runx2	AGTAAGAAGAGCCAGGCAGGTG	GTGTAAGTGAAGGTGGCTGGATAG
OSX	GATGGCGTCCTCTCTGCTT	TATGGCTTCTTTGTGCCTCC
ALP	AACCTGACTGACCCTTCCCTCT	TCAATCCTGCCTCCTTCCACTA
Col I	CTGCTGGCAAGAATGGCGA	GAAGCCACGATGACCCTTTATG
OCN	GCATTCTGCCTCTCTGACCTGG	GCTCCAAGTCCATTGTTGAGGTAG
β-actin	ACGGTCAGGTCATCACTATCG	GGCATAGAGGTCTTTACGGATG

### Statistical analysis

All quantitative experiments were representative of at least three independent samples and the result data were expressed as mean ± SD. The experiment for real-time polymerase chain reaction (RT-PCR) was operated in four replicates per group (*n* = 4). Statistical analysis was achieved according to a paired Student’s *t* test. The *P* values < 0.05 was considered statistically significant. For all figures, the following applies: * refers to *P* < 0.05 compared with BC group; δ refers to *P* < 0.05 compared with OC group.

## Results

### Structural characteristics of synthetic bone graft substitutes

**Figure 1:**
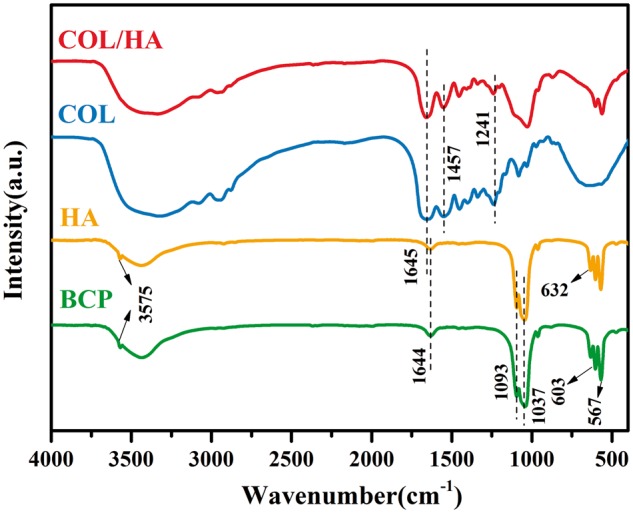
FT-IR Spectra of COL/HA, COL, HA and BCP

FT-IR spectra of four groups of synthetic bone graft substitutes are shown in Fig. 1. The characteristic absorptions of COL sample appears at 1645, 1457 and 1241 cm^−1^, attributable to amides I, II and III, respectively. The absorptions at 1093, 1037, 603 and 567 cm^−1^ are due to the stretching vibrations of ${\text{PO}}_{4}^{3}$^−^ in CaP [9, 46]. The absorption appeared at around 3450 cm^−1^ and 1644 cm^−1^ is arisen from the absorbed H_2_O in samples of HA and BCP. The characteristic adsorptions of both COL and phosphates appeared in COL/HA sample, confirming its composite nature [45]. In contrast, the characteristic absorptions of OH^-^ at 632 and 3575 cm^−1^ were present in HA and BCP samples, but absent in COL/HA. The recording of both OH^-^ characteristic adsorptions confirm the highly ordering of CaP lattice [47].

**Figure 2:**
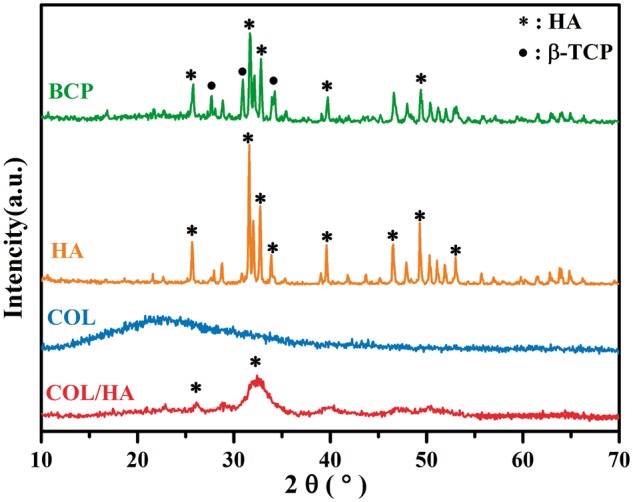
X-ray diffraction patterns of BCP, HA, COL and COL/HA. The reflections denoted by * and • ascribe to HA and β-TCP, respectively

The phase composition of these materials were analyzed by using XRD analysis, and their XRD patterns are given in Fig. 2. Strong reflections were recorded only in both bioceramic samples, in comparison with COL-based samples. The diffraction peaks at 2θ = 26.0°, 31.8°, 39.7° and 49.4° belong to the (002), (211), (310) and (213) planes of HA, respectively [48]. The reflections of β-TCP appear at 2θ = 31°, 27.5° and 34.5° [49]. In contrast, the apatite formed in COL/HA sample was indicated by a relatively strong peak at 2θ = 31.8°. The reflections denoted by * in XRD patterns of COL/HA are ascribed to HA. Our previous studies confirmed that a bone-like nature was achieved in COL/HA [8, 45]. In the case of COL sample, only a broad halo was recorded.

**Figure 3:**
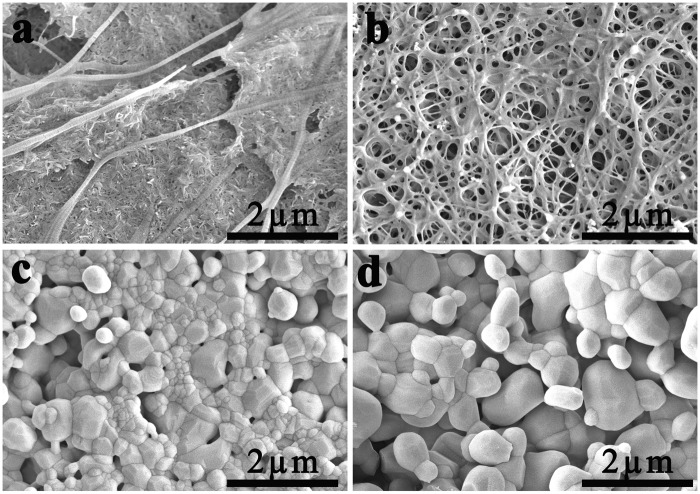
FE-SEM images of **(a)** COL/HA, **(b)** COL, **(c)** HA and **(d)** BCP

FE-SEM images of COL/HA, COL, HA and BCP are shown in Fig. 3. In the case of COL/HA, long fibers of the mineralized COL are observed to stretch across the sample, and many nano-sized needle-like objects are embedded among them (Fig. 3a) [29, 31]. Different from COL/HA, the COL gives a fibrous network due to the self-assembly of COL (Fig. 3b). In contrast to the fibrous structure of both COL/HA and COL, a particulate microstructure is prevalent in both HA and BCP. As both samples were sintered at 1100°C, polygonal crystals exist on the surfaces of both HA (Fig. 3c) and BCP (Fig. 3d) bioceramics. The grain size in HA ranges from 0.17 to 0.8 µm whereas that in BCP ranges from 0.3 to 1.8 µm [50].

### Cell proliferation

**Figure 4:**
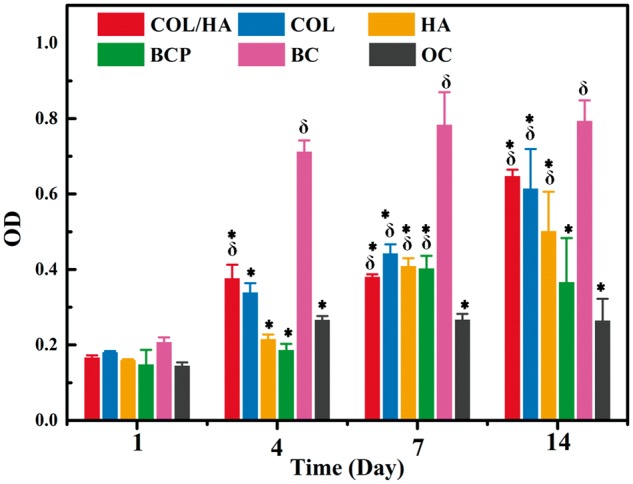
MTT Assays of the proliferation of rat BMSCs cultured for 1, 4, 7 and 14 days onto four groups of materials (COL/HA, COL, HA, BCP) as well as two groups of controls (BC and OC). BC and OC showed cells grown respectively in BM and osteogenic medium without the intervention of any materials. *refers to *P* < 0.05 compared with BC group, δ refers to P < 0.05 compared with OC group

The MTT data of the proliferation of rat BMSCs cultured for 1, 4, 7 and 14 days are given in Fig. 4. It was evident that cells onto four groups of materials gradually proliferated with increasing culture time. But, the proliferation tendency was different between COL-based samples and ceramic samples. Cell proliferation showed no difference on Day 1, but a higher proliferation rate was achieved onto COL/HA and COL than onto HA and BCP on Day 4. These differences in proliferation of cells onto four groups of materials disappeared on Day 7. The maximal proliferation was achieved on Day 14. In contrast, the cells in two groups of controls had the highest and poorest proliferation rate in BC and in OC, respectively, at each culture interval.

### Cell spreading morphology

**Figure 5:**
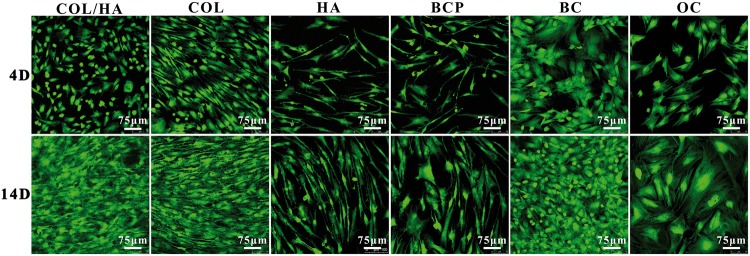
Confocal micrographs stained with FDA showing the growth and attachment of rat BMSCs after culturing for 4 and 14 days onto four groups of materials (COL/HA, COL, HA, BCP) as well as coverslips as two groups of controls (BC, OC)

The cells grown onto four groups of synthetic materials as well as coverslips as two groups of controls (BC and OC) were also stained by fluorescent dyes, and then imaged on a CLSM to reveal cell growth and spreading morphology. Confocal micrographs in Fig. 5 were the living cells stained with FDA after culturing for 4 and 14 days, showing that the number of living cells in all the six groups significantly increased on Day 14. Among six groups, relatively fewer cells grew on HA, BCP and OC. These results were in agreement with the MTT data of cell proliferation (Fig. 4). In terms of cell morphology, differences existed in different groups. Without the intervention of any materials, cells cultured in osteogenic medium (OC group) were larger and presented a cuboidal and even polygonal shape; cells cultured in BM (BC group) preferred to gather together. Among four groups of materials, cells onto COL-based materials were confluent while COL showed an obvious tendency to grow in an orientation way. In comparison, cells grown onto HA and BCP ceramics were relatively sparse in number but increase with a longer time (on Day 14). Cells exhibited as an elongated fusiform morphology especially in HA, and a polygonal shape was also observed in BCP.

**Figure 6:**
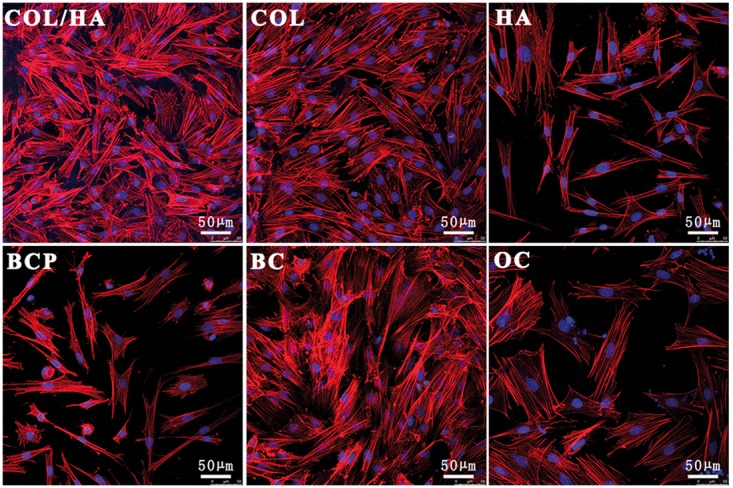
Representative fluorescence images of actin cytoskeleton and nuclei for rat BMSCs after culturing for 4 days onto four groups of materials (COL/HA, COL, HA, BCP) as well as coverslips as two groups of controls (BC, OC). Actin was stained red with TRITC-phalloidin and nucleus was stained blue with hoechst 33342

The rat BMSCs after culturing for 4 days were stained by TRITC-phalloidin for F-actin in cytoskeleton and by Hoechst 33342 for nucleus to confirm morphological differences mentioned earlier, and the fluorescence images are shown in Fig. 6. In general, the number of cells in ceramic groups (HA and BCP) and OC was much fewer than in COL/HA, COL and BC and distinct spreading morphologies could be easily discerned from them. Cells in OC exhibited a cuboidal shape and these cuboid-shaped cells were the largest in size among those cells in all the groups, indicated by the stress fibers of F-actins. In both ceramic groups, the presence of elongated fusiform cells was unique, but polygonal cells also could be found. In the cases of COL/HA and COL groups, much more cells were observed, spreading in either cuboidal or polygonal shape.

**Figure 7:**
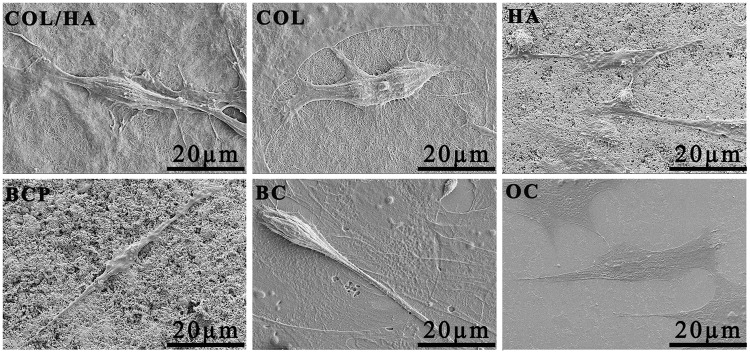
FE-SEM images of rat BMSCs after culturing for 4 days onto four groups of materials (COL/HA, COL, HA, BCP) as well as coverslips as two groups of controls (BC, OC). BC and OC showed cells grown respectively in BM and osteogenic medium without the intervention of any materials

Figure 7 is FE-SEM images of typical morphologies of rat BMSCs after culturing for 4 days onto four groups of materials as well as coverslips as two groups of controls (BC and OC). In either COL/HA or COL, a dense extracellular matrix associated with the cell layers was observed. The pseudopodia of a spindle-like cell were found to stretch long out. The cells grown on HA and BCP ceramic presented a flat polygonal and an extremely elongated shape, laying flatly on the surface composed by nano-sized particulates. The cell cultured on coverslips in BM alone (BC group) had a fusiform morphology. In OC group, the cuboid-like cells flatly spread on the coverslip, but the spreading area of each cell was much larger than the cell either on HA or on BCP and were consistent with the preceding results.

### Expression of osteogenic genes

To assess the osteogenic capability of rat BMSCs grown onto COL/HA, COL, HA and BCP, cells were cultured for 1, 4, 7, 14, 21 and 28 days, the expressions of transcription factors (RUNX-2 and OSX) and osteogenic marker genes (ALP, COL-I and OCN) were analyzed by RT-PCR. Cells grown in BM and osteogenic medium gave rise to two groups of controls, the BC and OC), without the intervention of any materials. At each culturing interval, six groups of average RT-PCR data were acquired, these normalized data vs. time were expressed as histograms and line charts. The histograms showed the differences among the groups while line charts presented the developmental trends of each group with time. In line charts, the area between the data of OC and BC were delimited as a reference model for comparing the osteogenic differentiation of cells onto different materials.

**Figure 8:**
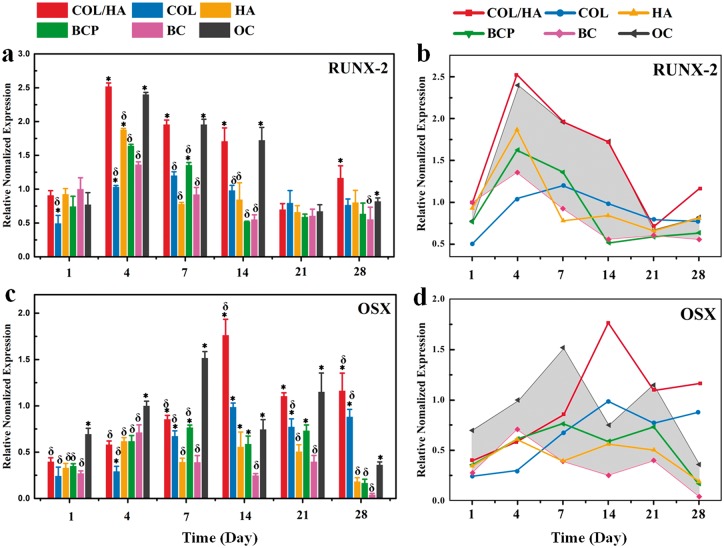
Expression of transcription factors for rat BMSCs after culturing for 1, 4, 7, 14, 21 and 28 days onto four groups of materials (COL/HA, COL, HA, BCP) as well as coverslips as two groups of controls (BC, OC), including **(a, b)** RUNX-2 and **(c, d)** OSX. In the line charts of (b) and (d), the shadow area as a reference model is delimited between the data of OC (the upper line) and BC, facilitating to compare the expression level of transcription factor of cells on different materials. * refers to P < 0.05 compared with BC group, δ refers to *P* < 0.05 compared with OC group

The expression levels of transcription factors, RUNX-2 and OSX, are shown in Fig. 8. Overall, the expression level of RUNX-2 displayed the trend of an early increment to the maximum and a subsequent slow decrement with a longer culturing interval (Fig. 8a and b). But, according to the reference model as delimited with a shadow area between the data of OC and BC, the difference in the expression level of RUNX-2 existed among four groups of bone grafting materials. A rapid elevation of the RUNX-2 expression to the apex occurred at Day 4 in all the samples except COL. The value of the apex level decreased in the order of COL/HA, OC, HA, BCP and BC. The apex for COL was achieved at Day 7. The histogram and line chart for the expression of OSX are given in Fig. 8c and d. It is obvious that the expression of OSX was much slower than the expression of RUNX-2. In the case of OC, the apex appeared at Day 4 for RUNX-2, and at Day 7 for OSX. The expression of OSX for HA and BCP followed the similar trend with OC. In COL/HA and COL, a steady elevation of OSX expression was observed, and this increase reached up to the maximum at Day 14 for COL/HA among six groups.

**Figure 9:**
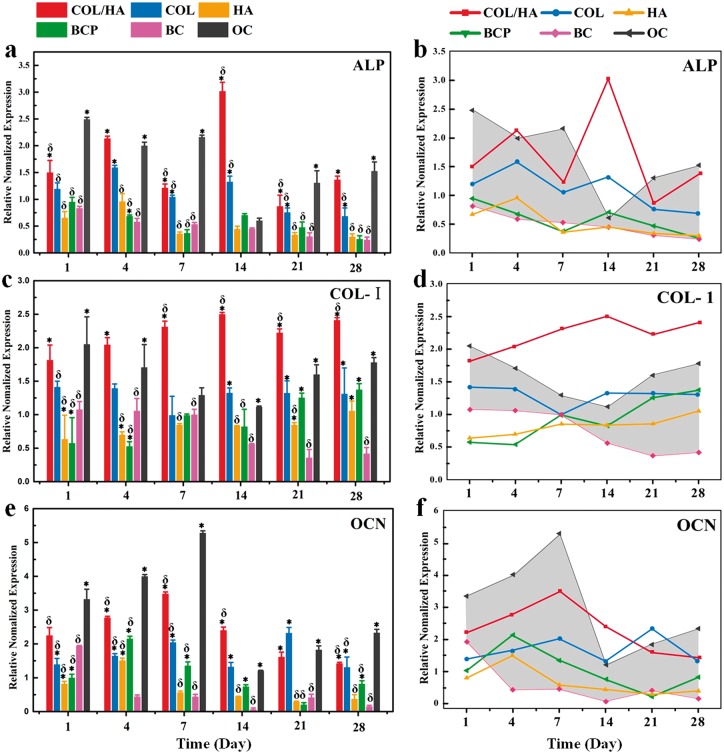
Expression of osteogenic genes for rat BMSCs after culturing for 1, 4, 7, 14, 21 and 28 days onto four groups of materials (COL/HA, COL, HA, BCP) as well as coverslips as two groups of controls (BC, OC), including **(a, b)** ALP, **(c, d)** COL-I and **(e, f)** OCN. In the line charts of (b), (d) and (f), the shadow area as a reference model is delimited between the data of OC (the upper line) and BC, facilitating to compare the expression level of osteogenic gene of cells on different materials. * refers to *P* < 0.05 compared with BC group, δ refers to *P* < 0.05 compared with OC group

The expression levels of osteogenic genes of ALP, COL-I and OCN are given in Fig. 9. For ALP, rat BMSCs grown onto all the materials, especially COL/HA and OC, exhibited a relatively high expression level at early stage during cell culture, i.e. at Days 1, 4 and 7. The COL/HA gave the highest expression level of ALP at 14th day, and the COL ranked the second. There existed no significant difference in the expression level of ALP among the groups of HA, BCP and BC (Fig. 9a and b). The expression of COL-I by rat BMSCs was found to enhance in COL/HA, COL and OC over the whole culturing time, and in BCP and HA a gradual increase trend was observed. A much higher value for COL-I expression in COL/HA was recorded among all the groups at each culturing intervals (Fig. 9c and d). The expression of OCN in OC, COL/HA, HA and BCP quickly increased and the maximal value reached at Day 4 for HA and BCP, and at Day 7 for OC, COL/HA. The apex value decreased in the order of OC, COL/HA, COL, BCP and HA (Fig. 9e and f).

## Discussion

Osteogenesis is a complex process substantially involving the differentiation of MSCs along osteoblast lineage through several maturation stages in an ordered time sequences and finally into the full active osteoblast phenotype. In this process several specific factors of osteogenesis would be highly associated, such as transcription factors and osteogenic genes. Transcription factors are nuclear proteins [51] serving as the ‘molecular switches’ to convert a stem cell into a terminal differentiate cell, and their expression levels are found to be sensitive to the properties of materials [52]. The master transcription factors of osteogenic differentiation in MSCs are considered to be RUNX-2 and OSX [53, 54]. The expression of RUNX-2 would be up-regulated in differentiating MSCs into immature osteoblasts, and decreased during their maturation. In contrast, the expression of OSX would be up-regulated and this is essentially needed during the maturation of osteoblast [55]. The expression of OSX was found to be activated by RUNX-2, but not exclusively dependent upon RUNX-2 activity [56]. The osteogenic differentiation of MSCs is a multi-step process [51], and during this process several specific genes would be highly expressed and regulated by transcription factors. For example, ALP and COL-I are considered as early markers [57] while OCN is the very late and specific marker of osteogenic differentiation [56].

The proliferation, morphology and osteogenic differentiation of MSCs could be influenced by growth factors [21, 22], biomaterials [6, 15, 16, 18–20] and/or bioactive molecules [34, 35, 58, 59]. In terms of developing materials for bone grafting, materials with composition and structure analogous to bone were reported to have an enhanced effect on the osteogenic capacity of cells [60, 61]. In this study, COL, COL/HA, HA and BCP were prepared as representative four groups of bone grafting materials. According to the MTT assays (Fig. 4), rat BMSCs proliferated well on all the four groups of materials although a higher proliferation rate was recorded at the early stage of culturing on both COL-based samples (COL and COL/HA) [62]. Different from the previously reported studies [25, 27, 29–31], two groups of controls (BC and OC) were applied in this work as a reference model. With the aid of this reference model which showed and thus delimited the basic and osteogenic behaviors of rat BMSCs without the intervention of any materials, it is convenient to acquire the key cellular phenomena in the osteogenic differentiation process and to compare the rate of the osteogenic capacity of cells.

When the growing behaviors of rat BMSCs were taken into account, including proliferation, cellular morphology and expression of specific osteogenic genes, the spreading morphology of cells was shown to have much closer relationship with osteogenic differentiation than the proliferation rate. BMSCs grown in BC group show the highest proliferation rate among all the groups but a quite low expression level of RNUX-2, OSX, ALP, COL-I and OCN (Figs 4, 8 and 9). Instead, a high expression level of these specific gene factors was recorded in the cells of OC group with almost the lowest proliferation rate. These fewer but larger cells presented a cuboidal shape, indicative of an osteoblastic lineage (Figs 5–7) [35]. Other typical spreading morphologies of cells onto four groups of materials also include polygonal and extremely elongated shapes. The latter was observed only in ceramic groups, especially in HA group.

The contrastive difference in the expression of these specific genes validates that to set two groups of controls as reference model is reasonable for evaluating the osteogenic activity of cells on materials. Indeed, the normalized RT-PCR data of cells in four groups of materials were almost fallen within the shadow area of this reference model delimited between BC and OC, as shown in the line charts of Figs 8 and 9. The expression level and time-dependent evolutional fashion varied in a specific gene among different groups. The definite expression further differed in the activation time and expression level of a gene. Meanwhile, this study also revealed that the expression of transcription factors occurred in an ordered time sequence, i.e. the expression of RUNX-2 was followed by OSX expression. Overall, rat BMSCs showed the highest osteogenic differentiation capability when cultured in COL/HA, as revealed by the quick activation and highest expression of RUNX-2, the highest expression of OSX and ALP, and especially high expression of COL-1. The excellent performance of COL/HA, i.e. mineralization of COL, in the osteogenic differentiation of MSCs was supported by previously reported studies [29, 43]. The early activation of RNUX-2, OSX and OCN was recorded in both ceramic groups, but more expression maximums of specific genes, i.e. OSC, COL-I and OCN of five genes, appeared in BCP groups. Meanwhile, the present results also indicated that COL was favorable to proliferation and osteogenic differentiation of BMSCs.

Obviously, the different osteogenic differentiation capability of cells onto four groups of materials was ascribed to the respective structural characteristics of COL, COL/HA, HA and BCP. The differences in microstructure and stiffness were obvious. Both COL and COL/HA were nanofibrous through reconstitution of COL gave rise to stronger fibrils than COL molecules or gelatin. Sintered HA and BCP were ceramic products with high elastic modulus, and their microstructures were composed by particulates. In addition, the easier dissolution may occur either in BCP or in COL/HA. According to the results above, this study demonstrated that COL/HA as an analogue to bone offers the most appropriate microenvironment for better promoting osteogenic differentiation of rat BMSCs [31, 44, 63, 64]. Finally, a reference model was put forth in this study by using two groups of controls. But, it is still expected that more materials or materials with changeable parameters will be used to verify the effectiveness and accuracy of this reference model for elucidating the data of cell experiments.

## Conclusion

Four kinds of bone constituent related biomaterials were prepared and characterized in this work. The cytocompatibility and osteogenesis capability were comparatively studied using rat BMSCs *in vitro*. MSCs cultured in TC plate with BM and standard osteogenic medium were used as the BC and the OC which could be used as a reference model applicable to comparing the growth behaviors of MSCs cultured on different materials. This study demonstrated that all the prepared materials have good cytocompatibility and can promote the osteogenic differentiation of rat BMSCs. COL/HA composites, COL and BCP have better ability in promoting the osteogenic differentiation of rat BMSCs compared with HA. Particularly, the MSCs in COL/HA group exhibited comparable up-regulation in osteogenic gene makers to OC group but had much better cell proliferation. Therefore, COL/HA composite can effectively promote osteogenic differentiation of MSCs and is a promising substitute material in bone tissue engineering.

## Funding

This work is supported by the Sichuan Provincial STP (No. 2017JY0018) and the National Basic Research Program of China (973 Program, No. 2012CB933600).

*Conflict of interest statement*. None declared.
